# Soluble cytotoxic T-lymphocyte–associated antigen 4 (sCTLA-4) as a potential biomarker for diagnosis and evaluation of the prognosis in Glioma

**DOI:** 10.1186/s12865-021-00422-y

**Published:** 2021-05-18

**Authors:** Jiajia Liu, Xiaoyi Tian, Yan Wang, Xixiong Kang, Wenqi Song

**Affiliations:** 1 Department of Clinical Laboratory Center, Beijing Children’s Hospital, Capital Medical University, National Center for Children’s Health, Beijing, 100045 China; 2 Laboratory Diagnosis Center, Beijing Tiantan Hospital, Capital Medical University, Beijing, 100050 China

**Keywords:** Soluble cytotoxic T-lymphocyte antigen 4(sCTLA-4), Glioma, Biomarker, Prognosis

## Abstract

**Background:**

The cytotoxic T-lymphocyte-associated antigen 4 (CTLA-4) is widely considered as a pivotal immune checkpoint molecule to suppress antitumor immunity. However, the significance of soluble CTLA-4 (sCTLA-4) remains unclear in the patients with brain glioma. Here we aimed to investigate the significance of serum sCTLA-4 levels as a noninvasive biomarker for diagnosis and evaluation of the prognosis in glioma patients.

**Methods:**

In this study, the levels of sCTLA-4 in serum from 50 patients diagnosed with different grade gliomas including preoperative and postoperative, and 50 healthy individuals were measured by an enzyme-linked immunosorbent assay (ELISA). And then ROC curve analysis and survival analyses were performed to explore the clinical significance of sCTLA-4.

**Results:**

Serum sCTLA-4 levels were significantly increased in patients with glioma compared to that of healthy individuals, and which was also positively correlated with the tumor grade. ROC curve analysis showed that the best cutoff value for sCTLA-4 for glioma is 112.1 pg/ml, as well as the sensitivity and specificity with 82.0 and 78.0%, respectively, and a cut-off value of 220.43 pg/ml was best distinguished in patients between low-grade glioma group and high-grade glioma group with sensitivity 73.1% and specificity 79.2%. Survival analysis revealed that the patients with high sCTLA-4 levels (> 189.64 pg/ml) had shorter progression-free survival (PFS) compared to those with low sCTLA-4 levels (≤189.64 pg/ml). In the univariate analysis, elder, high-grade tumor, high sCTLA-4 levels and high Ki-67 index were significantly associated with shorter PFS. In the multivariate analysis, sCTLA-4 levels and tumor grade remained an independent prognostic factor.

**Conclusion:**

These findings indicated that serum sCTLA-4 levels play a critical role in the pathogenesis and development of glioma, which might become a valuable predictive biomarker for supplementary diagnosis and evaluation of the progress and prognosis in glioma.

## Background

In the early 1900s, Paul Ehrlich first proposed that tumors would be more common without the protection of the immune system, which initiated a new era for the study of the relationship between the body’s immune system and tumors [1]. Accumulating reports have shown that the generation, development and prognosis of tumors are closely related to the body’s immunity, and immunotherapy plays an important role in killing tumor cells by enhancing the function of the immune cells or relieving the local immunosuppressive microenvironment [2, 3]. T cell-mediated immunity response have been confirmed to play a key role in the antitumor immunity, and the activation and proliferation of T cells are critical in this process [3, 4]. It is well known that T-cell-mediated immune response requires at least two signals from cell surface receptors: the first signal is the binding of antigen with TCR on T cells and then the reaction of T cells is finely regulated by the synergistic stimulating molecules and co-inhibitory molecules, so as to achieve moderate immune function, and these suppression signals are the checkpoints of immunity and also the key factor of immune escape [4, 5]. Cytotoxic T lymphocyte antigen-4 (CTLA-4, CD152) is widely considered as a pivotal coinhibitory molecule that suppressed antitumor immunity by down-regulating T-cell mediated immune responses [6].

CTLA-4, a transmembrane glycoprotein, is expressed on the surface of T cells and transmits an inhibitory signal to T cells [6]. CTLA-4 is highly homologous to the T-cell co-stimulatory protein as CD28, and shares two ligands including CD80 (B7–1) and CD86 (B7–2) on antigen-presenting cells (APCs), whereas the affinity of CTLA-4 for B7 are stronger than CD28 [7]. CTLA-4 binding to B7 blocks the interaction of B7 and CD28, and results in a negative signal, which suppresses the activation and proliferation of immune cell, production of cytokine and cell cycle progression [8].

The CTLA-4 protein is not only expressed on the cell surface, but also has a different form with soluble CTLA-4 (sCTLA-4) which is the product of transcriptional translation of CTLA-4 gene first discovered by Magistrelli in 1999 [9]. sCTLA-4, a 137-amino-acid polypeptide with complete function, is secreted by non-activated T cells and also can be detected in normal human serum [7–9]. Mature CTLA-4 molecules are composed of extracellular, transmembrane and cytoplasmic regions [8–10]. sCTLA-4 lacks transmembrane domain and secretes from the cells, although the amino acid residues of it binding to B7 remains intact [9, 10]. Analysis of human T cells in vitro has shown that sCTLA-4 secretion can increase during immune responses and has powerful inhibitory function, as isoform-specific blockade of sCTLA-4 significantly increases antigen-driven proliferation and cytokine secretion. Recent studies have revealed that sCTLA-4 is preferentially combined to B7 on APCs compared to CD28 on T lymphocytes, and plays a significant down-regulatory role [8–11]. Most of the studies on CTLA-4 have been committed to CTLA-4 membrane protein, especially in the field of cancer research, however, the gradual unraveling of the significant role of soluble form in immunosuppressive function is now attracting attention toward soluble form as well [2–4]. Studies have shown that high levels of sCTLA-4 were detected in patients with some autoimmune diseases, such as autoimmune thyroid disease [11, 12] systemic lupus erythematosus (SLE) [13, 14], type 1 diabetes mellitus [15], immune thrombocytopenia [16]. Markedly increased levels of sCTLA-4 in peripheral blood were observed in some cancers [17–19]. In addition, sCTLA-4 can neutralize the anti-CTLA-4-mAb in vivo. In peripheral blood mononuclear cell responses, the blockade of sCTLA-4 activates the proliferation of CD4^**+**^ and CD8^**+**^ T cells and promotes increased cytokine secretion, which enhances antitumor effects. However, no investigation is reported about the soluble CTLA-4 levels in blood circulation of the patients with brain glioma that is the most common type among primary tumors in central nervous system with rapid growth and poor prognosis. The main aim of this study is to explore the expression and clinical significance of serum sCTLA-4 in the patients with brain glioma.

## Material and methods

### Patients

**Fig. 1:**
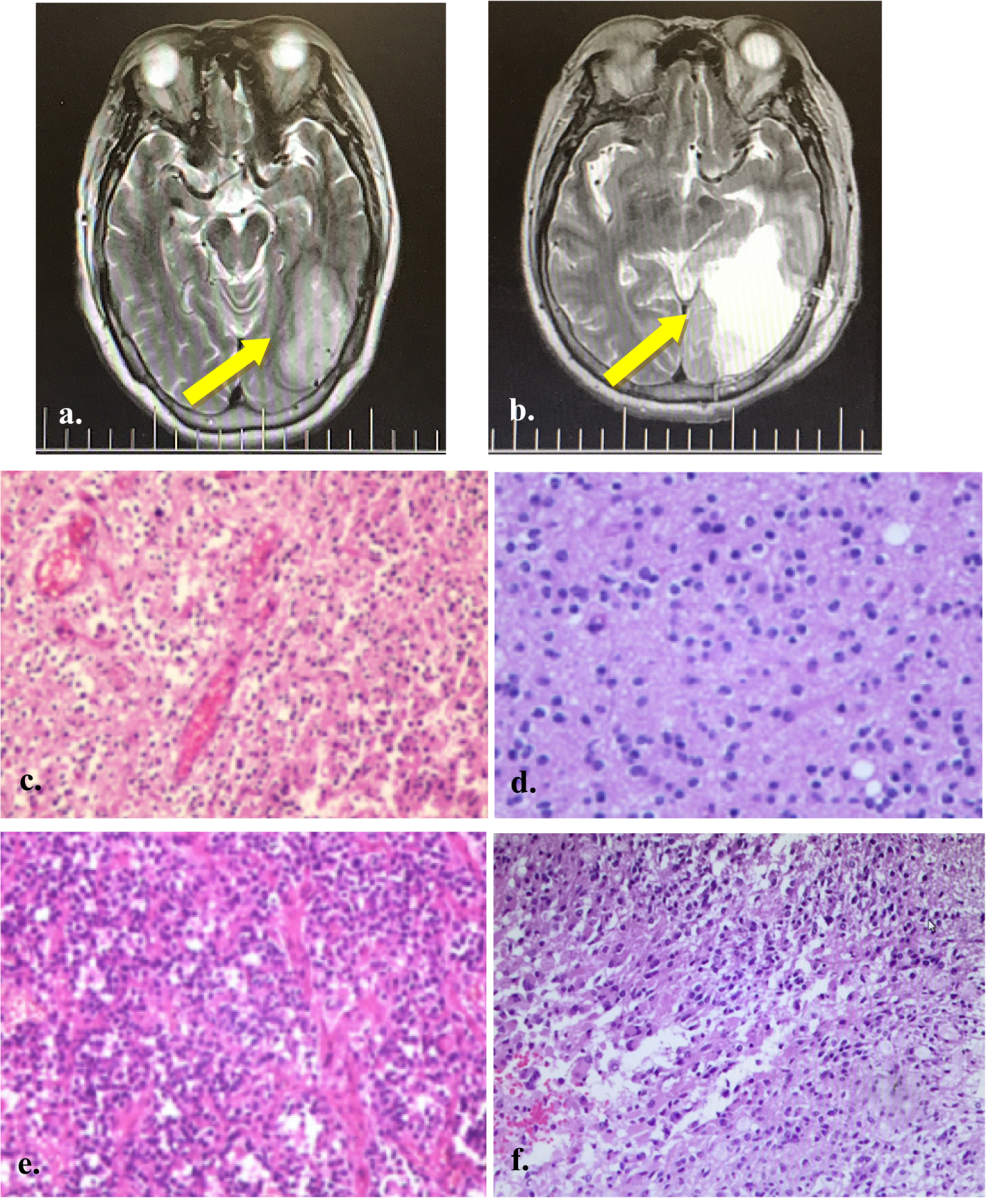
Magnetic resonance imaging (MRI) of a case of glioblastoma and histopathology of different grade gliomas. **a** Preoperative T2-weighted axial image showed left occipitotemporal tumor with solid. **b** Immediate post-operative (at 24 h) T2-weighted axial image. **c-f** Histopathology of different grade gliomas (WHO I-IV) (Hematoxylin and eosin staining, × 200)

In this study, 50 patients including 27 men and 23 women diagnosed with different grade gliomas and 50 age- and sex-matched healthy individuals without a personal or familial history of cancer or autoimmune diseases were recruited in Beijing Tiantan Hospital between October 2017 and January 2019. Eligibility criteria for patients as follows: (1) pathological glioma according to the World Health Organization (WHO) classification; (2) no other diseases such as autoimmune diseases, other types of tumors; (3) no history of any treatment such as tumor excision or drug therapy; (4) availability of complete clinical information and survival data. Figure 1 showed magnetic resonance imaging (MRI) of a case of glioblastoma and histopathology images of different grade glioma patients. Serum samples were collected before and after operation on the patients, and from healthy individuals, then stored at − 80 °C. The study was conducted in accordance with the Declaration of Helsinki in 1975 and the REMARK guidelines for biomarker studies. Signed informed consent was obtained from all participants. For the minors under the age of 18 years, informed consent has been obtained from a parent and/or legal guardian. This study was approved by the institutional research ethics committee of Beijing Tiantan Hospital.

### Soluble CTLA-4 measurement

The serum levels of CTLA-4 were measured by an enzyme-linked immunosorbent assay (ELISA) kit (Cusabio Biotech, Wuhan, China) according to the manufacturer’s instructions. All samples were tested in triplicate. Briefly, 100 μL of each diluted standards or samples were added to per well and incubated for 2 h at 37 °C. Add 100 μl of Biotin-antibody to each well and incubate with shaking for 1 h at 37 °C. Aspirate each well and wash, repeating the process two times for a total of three washes and then 100 μL of HRP-avidin was added. Add 90 μl of TMB Substrate to each well and incubate for 15–30 min at 37 °C. The reaction was stopped by addition of 50 μL of stop solution, and plates were read at 450/540 nm using an ELISA plate reader (Thermo Multiskan FC, Thermo Scientific, America). sCTLA-4 concentrations (pg/mL) in serum samples were calculated from a standard curve generated in the ELISA plate reader. The intra-assay and inter-assay coefficients of variation were below 10%. The minimum detectable level of sCTLA-4 is below 31.25 pg/ml.

### Statistical analysis

SPSS 21.0 software (SPSS Inc., Chicago, IL, USA) was used to perform data analysis. Serum CTLA-4 levels in the healthy controls and patients before and after operation were compared using the Mann–Whitney *U* test. Spearman’s analyses were performed to determine correlation of serum sCTLA-4 levels with clinicopathological parameters. ROC curve analysis was performed to determine the best positive sCTLA-4 reference value. Survival curve was performed using the Kaplan–Meier method and compared using log-rank test. Progression-free survival (PFS) was defined as the time between the first day of diagnosis and the date of disease relapse or progression. Both univariable and multivariable Cox proportional hazard regression analyses were used to examine the association between sCTLA-4 level and survival. *P* value< 0.05 was considered to be significant.

## Results

### Clinical characteristics of patients

The clinical characteristics of 100 participants consisted of 50 patients and 50 healthy individuals were showed in Table 1. All clinical data were obtained from electronic medical charts. 50 patients included 27 male patients and 23 female patients, and the median age of them was 50.5 years (range: 10–70 years). Twenty-four (48.0%) were diagnosed with low-grade gliomas (include stage I and stage II), and twenty-six (52.0%) with high-grade gliomas (include stage III and stage IV) according to WHO classification. Immunohistochemistry (IHC) measurement of all samples was independently performed by two pathologists. It was determined as positive if Ki-67 nucleus was stained. Negative (−) if the percentage of positive cells was lower than 5%, and weakly positive expression (+) for the percentage of positive cells lower than 10%, moderately positive expression (++) for the percentage of positive cells between 10 and 25%, strongly positive expression (+++) for the percentage of positive cells higher than 25%. All patients underwent surgical treatments.

**Table 1:** Characteristics of participants (*N* = 100)

Feature	No of cases (%)		Total
	Patients	Control	
Overall	50	50	100
Gender			
Female	23 (46.0)	24 (48.0)	47 (47.0)
Male	27 (54.0)	26 (52.0)	53 (53.0)
Age (years)			
≤ 60	31 (62.0)	27 (54.0)	58 (58.0)
> 60	19 (38.0)	23 (46.0)	42 (42.0)
Tobacco smoking			
Ever	17 (34.0)	19 (38.0)	36 (36.0)
Never	33 (66.0)	31 (62.0)	64 (64.0)
Alcohol drinking			
Ever	15 (30.0)	18 (36.0)	33 (33.0)
Never	35 (70.0)	32 (64.0)	67 (67.0)
Grade			
Low-grade glioma	24 (48.0)	–	–
High-grade glioma	26 (52.0)	–	–
Tumor site			
Frontal lobe	15 (30.0)	–	–
Temporal lobe	11 (22.0)	–	–
Parietal lobe	6 (12.0)	–	–
Occipital lobe	7 (14.0)	–	–
Other	11 (22.0)	–	–
Ki-67 labeling index			
Negative (−)	10 (20.0)	–	–
Weakly positive (+)	16 (32.0)	–	–
Moderately positive (++)	14 (28.0)	–	–
Strongly positive (+++)	10 (20.0)	–	–

### Serum levels of sCTLA-4

**Fig. 2:**
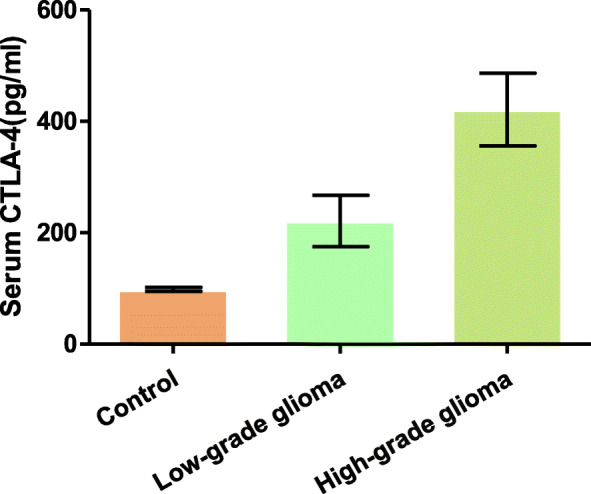
Serum sCTLA-4 levels in patients with glioma and in controls. Patients with glioma have a statistically higher concentration of circulating sCTLA4 than control subjects (*p <* 0.05). Median level of sCTLA-4 for the patients with high-grade glioma was higher than those in the low-grade glioma group and control subjects (all *p <* 0.05). sCTLA-4, soluble cytotoxic T-lymphocyte antigen 4

sCTLA-4 levels were detected by ELISA in the control population and patients. The results showed that the patients with glioma before therapy had a statistically higher concentration of sCTLA-4 than that of control subjects (median of 198.26 pg/ml versus 92.73 pg/ml, respectively, *p <* 0.05). Median level of sCTLA-4 for the patients with high-grade glioma was 320.16 pg/ml, which was higher than those in the low-grade glioma group (median of 137.04 pg/ml) and control subjects (median of 92.73 pg/ml) (*p <* 0.05; Fig. 2).

### The diagnostic value analysis of serum sCTLA-4 in glioma patients

**Fig. 3:**
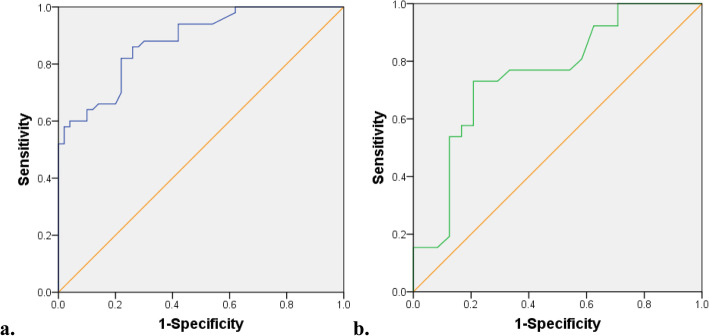
ROC curve analysis for serum sCTLA-4 concentration. **a** The most discriminative cutoff value for sCTLA-4 for glioma was 112.1 pg/ml with an AUC value of 0.880 (95% confidence interval: 0.816 to 0.943, *p* < 0.05). The sensitivity and specificity were 82.0 and 78.0%, respectively. **b** A cut-off value of 220.43 pg/ml was best distinguished in patients between low-grade glioma group and high-grade glioma group with sensitivity73.1% and specificity 79.2% (95% confidence interval: 0.619 to 0.894, *p* < 0.05). sCTLA-4, soluble cytotoxic T-lymphocyte antigen 4

To determine the best positive reference values of sCTLA-4 to improve its diagnostic value of glioma, ROC curve analysis was performed to predict the best cutoff value for sCTLA-4 for glioma, the value was 112.1 pg/ml with an AUC value of 0.880 (95% confidence interval: 0.816 to 0.943, *p* < 0.05). The sensitivity and specificity were 82.0 and 78.0%, respectively (Fig. 3a). The expression level of sCTLA-4 showed significant difference between low-grade glioma group and high-grade glioma group. A cut-off value of 220.43 pg/ml with an AUC value of 0.756 was best distinguished in patients between low-grade glioma group and high-grade glioma group (95% confidence interval: 0.619 to 0.894, *p* < 0.05), and the sensitivity and specificity were 73.1 and 79.2%, respectively (Fig. 3b).

### Correlation of serum sCTLA-4 levels with clinicopathological parameters in glioma patients

The correlation between concentration of sCTLA-4 and the clinicopathological parameters were analyzed (Table 2). The results demonstrated that sCTLA-4 level was positively correlated with the tumor grade, Ki-67 labeling index and WBC counts. There were no correlations between serum sCTLA-4 levels and other parameters including age, gender, tumor site, red blood cell (RBC) counts, platelet (PLT) counts, smoking history, drinking history (all *p* > 0.05).

**Table 2:** Correlation Between Serum sCTLA-4 and clinicopathological parameters

clinicopathological parameters	Correlation coefficient	*P*-value
Age	0.130	0.367
Gender	0.185	0.223
Smoking history	0.260	0.085
Drinking history	− 0.014	0.926
Tumor Grade	0.444	< 0.05
Tumor site	−0.150	0.298
Ki-67 labeling index	0.657	< 0.05
WBC Counts	0.361	< 0.05
RBC Counts	0.265	0.079
PLT Counts	0.088	0.565

Notes: Of all the parameters evaluated in this research, tumor grade, Ki-67 labeling index and WBC were correlated with sCTLA-4 level in patient group. *p < 0.05. *sCTLA-4* Soluble cytotoxic T-lymphocyte antigen 4, *WBC* White blood cell, *RBC* Red blood cell, *PLT* Platelet

### Survival analysis

**Fig. 4:**
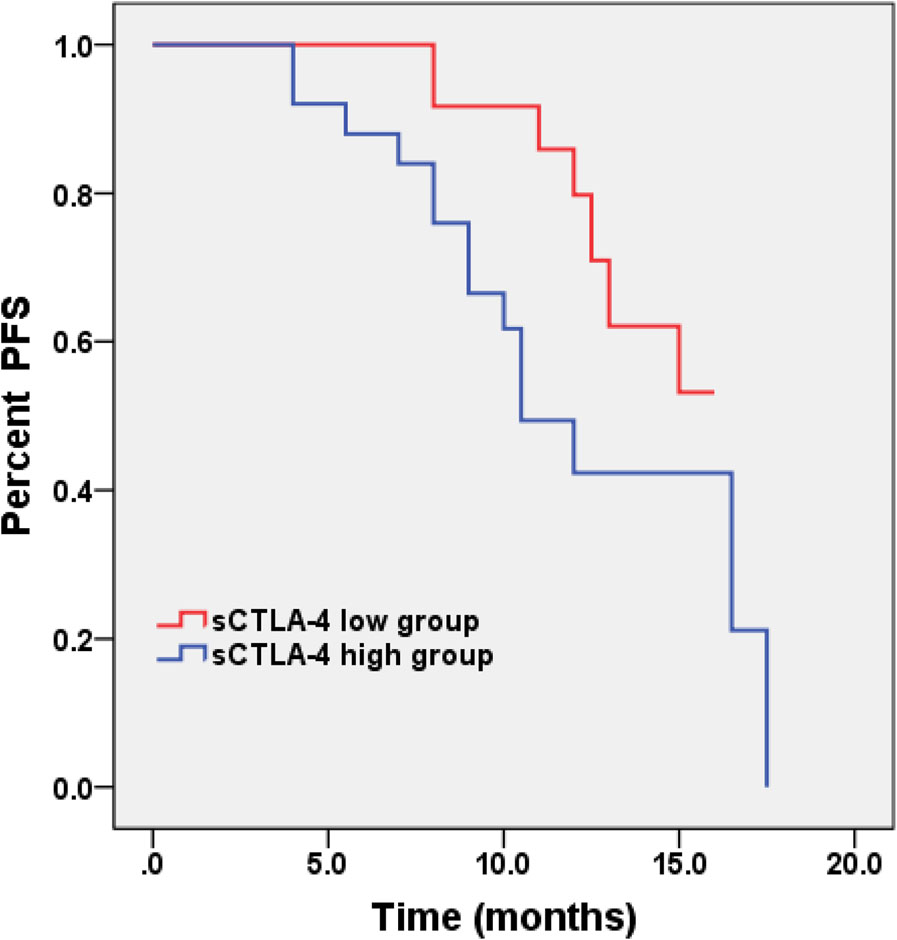
Survival curves for PFS for all patients with glioma. Survival analysis was according to sCTLA-4 levels during therapy, with blue indicating high sCTLA-4 levels (> 189.64 pg/ml) and red denoting low sCTLA-4 levels (≤189.64 pg/ml) (*p* < 0.05, log-rank test)

The patients were divided into two groups according to the median value of 189.64 pg/ml calculated by the sCTLA-4 levels after therapy. The median follow-up time was 11 months (4–18 months). Survival analysis revealed that the patients with high sCTLA-4 levels (> 189.64 pg/ml) had shorter progression-free survival (PFS) compared to those with low sCTLA-4 levels (≤189.64 pg/ml) (10 months vs. not reached, *p* < 0.05 log-rank test) (Fig. 4). Table 3 showed the results of the univariate and multivariate analyses associated with PFS. In the univariate analysis, elder, high-grade tumor, high sCTLA-4 levels and high Ki-67 index were significantly associated with shorter PFS (all *p* < 0.05). In the multivariate analysis, sCTLA-4 levels and tumor grade remained an independent prognostic factor (HR: 2.521, 95% CI: 1.012 to 6.280, *p* = 0.047; HR: 2.941, 95% CI: 1.037 to 8.336, *p* = 0.042, respectively).

**Table 3:** Univariate and Multivariate Analyses for PFS

Parameters	PFS		
	Univariate Analysis	Multivariate Analysis	
	*p*	HR (95%CI)	*p*
Age	< 0.05		0.128
Gender	0.939		
Tumor Grade	< 0.05	2.941 (1.037–8.336)	0.042
sCTLA-4 levels (> 189.64 pg/ml)	< 0.05	2.521 (1.012–6.280)	0.047
Ki-67 index	< 0.05		0.677

Note: *CI* Confidence interval, *HR* Hazard ratio

## Discussion

Gliomas are the most common type of brain primary malignancies that produce severe damage to the brain as well as with difficult diagnosis and poor prognostic outcome [20]. The World Health Organization has classified gliomas into 4 grades. Grades I and II are typically considered low-grade gliomas, and grade III and IV tumors are considered high-grade gliomas [21]. Gliomas have the characteristics of high incidence accounting for 35.2–61.0% of all primary brain tumors, high recurrence rate owing to their highly invasive potential, high mortality rate especially glioblastoma (GBM; grade IV) with a median survival of about 12 to 15 months [22, 23].

Traditional concept holds that the brain is the location of immune exemption owning to the existence of the blood-brain barrier (BBB), but the BBB is damaged under pathological conditions, which is more conducive to the transfer of immune cells [21–24]. Recent research has found that, like other tissues, the brain can connect to the peripheral immune system through the lymphatic vessels of the brain, helping to facilitate the tumor’s immune response [25]. These studies of the intracranial lymphatic system have challenged the classical dogma that the central nervous system is immune-privileged and lacks immunosurveillance, which also give the elicitation of the immunological checkpoint targeted therapy of glioma.

Tumor immunology is a hot topic in oncology, and immune checkpoint is particularly important in tumor immunology. CTLA-4 is widely considered as a pivotal immune checkpoint molecule in down-regulating T-cell mediated immune responses, suppressing antitumor effects [3, 4]. Most of the studies on CTLA-4 have been committed to CTLA-4 membrane protein, especially in the field of cancer research. Previous studies have investigated higher membrane-bound CTLA-4 (mCTLA-4) expression was found in patients with melanoma, chronic myelomonocytic leukemia, and acute myeloid leukemia [26, 27]. Fong et al. studied the expression of CTLA-4 on peripheral blood lymphocyte subsets in GBM patients treated with DC vaccination and found CTLA-4 can predict survival in GBM patients. Higher expression of CTLA-4 was correlated with shorter survival after treatment [28]. Liu et al. investigated the protein level of CTLA-4 in human glioma samples and found higher CTLA-4 expression was found in patients with higher grade gliomas than in patients with lower grade gliomas. Moreover, patients with lower CTLA-4 expression exhibited significantly longer overall survival [29]. These findings suggested that increased mCTLA-4 expression conferred a worse outcome in glioma.

The mRNA encoding sCTLA-4, one of the isoforms of CTLA-4, like membrane-bound CTLA-4 (mCTLA-4) can bind to the B7 ligands, and interfere with their ligation to CD28, thus inhibiting T-cell responses [6–8]. In addition, sCTLA-4 can neutralize the anti-CTLA-4-mAb in vivo. In peripheral blood mononuclear cell responses, the blockade of sCTLA-4 activates the proliferation of CD4^**+**^ and CD8^**+**^ T cells and promotes increased cytokine secretion, which enhances antitumor effects. We previously demonstrated abnormal expressions of sCTLA-4 in breast cancer, liver cancer and NSCLC, suggesting that serum sCTLA-4 may play an important role in the immune microenvironment of cancer [19, 30, 31]. Many studies regarding tumors are ongoing, and the relevance of soluble receptors and ligands to various tumors is becoming increasingly apparent. As soluble molecule, its serum levels can be easily detected. sCTLA-4 may also be critical factors for evaluating the severity and prognosis of cancer and many other diseases since most patients experience changes. However, the expression of the sCTLA-4 levels in blood circulation of the patients with brain glioma has not been investigated, and the biological significance of sCTLA-4 has not been fully elucidated in glioma. This study focused on evaluating the value of serum sCTLA-4 for diagnosis and prognosis estimating among cases with glioma. In this study, the significantly higher sCTLA-4 level was found in serum from the patients with glioma than controls. And serum sCTLA-4 levels were positively correlated with the tumor grade. Further ROC curve analysis, the best cutoff value for sCTLA-4 for glioma is 112.1 pg/ml, as well as the sensitivity and specificity with 82.0 and 78.0%, respectively, and a cut-off value of 220.43 pg/ml was best distinguished in patients between low-grade glioma group and high-grade glioma group with sensitivity73.1% and specificity 79.2%, suggesting that sCTLA4 had clinical value for the auxiliary diagnosis and grading of glioma. It is not clear about elevated circulating levels of sCTLA-4 in many patients with glioma, and increased concentrations of serum sCTLA-4 have also been reported in patients with other types of tumors [24–28]. Erfani et al. discovered significantly higher levels of sCTLA-4 in patients with breast cancer than those of healthy controls [19]. In a study by Roncella et al., it can be concluded that in both serum and pleural effusion sCTLA-4 levels were higher in malignant pleural mesothelioma as compared to pulmonary benign disease [32]. sCTLA-4 might be primarily produced and released by T lymphocytes. A study in brain glioma patients indicated that CTLA-4 is highly expressed on T cells, specifically the effector CD4 + T cells. More importantly, the expression of CTLA-4 is correlated with the glioma grade [28]. Now, the critical question is whether increased sCTLA-4 levels are beneficial or harmful to the patients with glioma. We investigated correlation between serum levels of sCTLA-4 in glioma patients with survival outcomes. We found PFS was significantly shorter for patients with an increased serum sCTLA-4 concentration, suggesting that sCTLA-4 might be a prognostic factor for glioma. Similar findings have been made in many reports that sCTLA-4 might be a prognostic factor for various malignant tumors [19, 28, 30–32]. The mechanisms by which increased serum sCTLA-4 levels contribute to poor prognosis in glioma are not clear. Many researchers hold that sCTLA-4 might compete with CD28 for B7 interfering with B7-CD28 ligation, thus blocking T cell co-stimulation just like the membrane-bound form, which would inhibit the effect of antitumor immunity, leading to failure of the therapy. On the other hand, some reports have been demonstrated that sCTLA-4 is able to bind to APCs as well and inhibit the expression of CD80/CD86 through a process called transendocytosis [11, 27, 33].

Further studies are needed to investigate the role of sCTLA-4 in the pathogenesis and development of gliomas. Given CTLA-4 complex mechanism on the immune system, inhibiting CTLA-4 action might enhance immunosurveillance, and first antibody against CTLA-4, ipilimumab, which can bind both mCTLA-4 and sCTLA-4, was approved in 2011 for the treatment of melanoma [27]. The rationale of anti-CTLA-4 antibody therapy is that it enhances immune responses against tumor by enhancing tumor-specific effector T-cell response [33]. The clinical success of antibodies targeting CTLA-4 marks a significant milestone as these agents established immunotherapy as an emerging therapeutic modality of tumor treatment strategies next to surgery, chemotherapy, and radiation therapy. However, the clinical response to checkpoint blockade therapy can vary in different tumor types and individuals, and many works have been directed toward finding predictive biomarkers to help identify patients who will get the most benefit from these therapies [34]. Some studies have shown that blood sCTLA-4 levels might be used as a promising predictive biomarker for ipilimumab therapy for NSCLC and melanoma [34, 35]. In contrast, few research in this field of glioma is reported due to the complexity and particularity of central nervous system. Our conclusions are limited, and future studies should be conducted in a larger sample of patients to analyze the dynamic change of sCTLA-4 as a prognostic or predictive biomarker of traditional treatments as well as immune checkpoint inhibitors.

## Conclusion

In conclusion, it is the first time to show the relationship between serum sCTLA-4 levels and glioma including diagnosis and disease progression. Serum sCTLA-4 is a valuable and less invasive biomarker in clinical practice, and may be an important prognostic factor for this disease, which offers new insight into potential therapeutic strategies. Better designed studies in the future are likely to have a larger sample size and a longer follow-up period and dynamic analysis of the changes of sCTLA-4 in serum might be helpful in evaluating the progression and prognosis of glioma.

## Data Availability

The datasets used and/or analyzed during the current study available from the corresponding author on reasonable request.

## Abbreviations

sCTLA-4: Soluble Cytotoxic T lymphocyte antigen-4
ELISA: Enzyme-linked immunosorbent assay
PFS: Progression-free survival
APCs: Antigen-presenting cells
MRI: Magnetic resonance imaging
